# 

**Published:** 1789

**Authors:** 


### CONTENTS.

Page

I. *AN Account of a remarkable Disease of the Heart, Lungs, and one of the external Mammæ; with the morbid Appearances as they presented on Dissection. By Mr.* Robert Kinglake, *Surgeon at Chipping-Norton in Oxfordshire.* — — 341

II. *Facts relative to the Small Pox. By Mr.* Thomas Davidson, *Surgeon in Carriacou.* — — — — — 353

III. *An Account of the good Effects of Mercury in two Cases of impeded Deglutition; to which is added an Instance of the Relief obtained from the same Remedy in a spasmodic Affection of the Neck of the Bladder. By Mr.* Samuel Patten, *Surgeon in London.* — 356

IV. *Observations on Pemphigus. By Mr.* Thomas Christie, *Member of the Medical and Antiquarian Societies of Edinburgh.* 361

V. *A critical and anatomical Examination of the Parts immediately interested in the Operation for a Cataract; with an Attempt to render the Operation itself, whether by Depression or Extraction, more certain and successful. By* Silvester O'Halloran, *Esq. M.R.I.A.*

Page

*Honorary Member of the Royal College of Surgeons in Ireland, and Surgeon to the County of Limerick Hoſpital. — From The Tranſactions of the Royal Iriſh Academy.* — — 395

VI. *An Account of a Monſter of the human Species, in two Letters; one from Baron* Reichel *to Sir* Joſeph Banks, *Bart., and the other from Mr.* James Anderſon *to Baron* Reichel.— *From the Philoſophical Tranſactions of the Royal Society of London.* — 422

Catalogue *of* Books. — — 426

Index. — — — 435

